# Estimated effects of onset of dementia on labor market

**DOI:** 10.1002/alz70860_103216

**Published:** 2025-12-23

**Authors:** Maria Prados, Carlos Acosta, Soeren Mattke

**Affiliations:** ^1^ University of Southern California, Los Angeles, CA, USA; ^2^ Biogen, Baar, zug, Switzerland

## Abstract

**Background:**

Treatments for secondary prevention of Alzheimer's disease (AD), aimed at delaying progression from preclinical to prodromal AD, are in late‐stage clinical trials. Given their use in cognitively unimpaired and younger populations, these treatments might maintain productivity and workforce participation. Here, we estimate their potential influence on the labor market for the United States (US) population.

**Method:**

We used longitudinal data from the Health and Retirement Study, a biennial nationally representative survey of US adults 50+, from 1996 to 2020. We estimated, by gender, the differential change in labor force participation, annual labor earnings, and social assistance payments between incident cases of cognitively impaired patients, using the Langa‐Weir classification, and statistically matched cognitively normal counterparts.

**Result:**

We identified 7,636 incident cases of mild cognitive impairment (MCI) and dementia out of 21,087 respondents aged 50‐79, who were cognitively normal and participating in the workforce in the prior wave of the survey. Both genders were ∼5 percentage points (pp) less likely to participate in the workforce after onset of cognitive impairment than matched controls. The average reduction in annual earnings was $4,466 (8.5%) for men and $1,574 (5.2%) for women. Incident MCI/dementia was significantly associated with increased receipt of social assistance benefits (2.9 pp for men, 3.75 pp for women) but not with the benefit amount. As around half of these cases are due to AD, these estimates imply that incident prodromal AD is associated with an aggregate loss of approximately $896 million in labor earnings annually ($667 million to male and $229 million to female workers). The estimated effect on the labor force was a reduction of 14,698 workers (8,005 male and 6,693 female) and on social assistance rolls an increase of around 9,787 recipients.

**Conclusion:**

Onset of cognitive impairment was associated with statistically significant and meaningful declines in workforce participation and labor income and increased participation in social security programs in a representative sample of U.S. adults. As it is estimated that ∼half of those cases are due to AD, a treatment that delays transition from preclinical to prodromal disease could generate substantial economic value.

